# The optimal dose of succinylcholine for rapid sequence induction: a systematic review and meta-analysis of randomized trials

**DOI:** 10.1186/s12871-020-00968-1

**Published:** 2020-03-02

**Authors:** Alessandro Putzu, Martin R. Tramèr, Maxim Giffa, Christoph Czarnetzki

**Affiliations:** 1 Division of Anaesthesiology, Department of Anaesthesiology, Pharmacology, Intensive Care and Emergency Medicine, Geneva University Hospitals, 4 Rue Gabrielle Perret-Gentil, 1205 Geneva, Switzerland; 2 Faculty of Medicine, Geneva University, Geneva, Switzerland; 3 Division of Anaesthesiology, Ospedale Regionale di Lugano – Civico, Lugano, Switzerland

**Keywords:** General anaesthesia, Tracheal intubation, Succinylcholine, Suxamethonium

## Abstract

**Background:**

The evidence base for the widely accepted standard regimen of succinylcholine for rapid sequence induction (1.0 mg kg^− 1^) remains unclear.

**Methods:**

We performed a systematic review and meta-analysis of randomized trials comparing any succinylcholine regimen with the standard regimen (1.0 mg kg^− 1^) and reporting on intubating conditions and/or apnoea times. Results were expressed as absolute risk differences (ARD) for dichotomous data and mean differences (MD) for continuous data.

**Results:**

We retrieved six trials with relevant data of 864 patients (ASA 1 or 2, aged 18–65 years, body mass index < 30 kg m^− 2^). Four regimens (0.3, 0.4, 0.5, 0.6 mg kg^− 1^) were compared with 1.0 mg kg^− 1^ in at least three trials each, and three (0.8, 1.5, 2 mg kg^− 1^) in one each. With 0.3 to 0.5 mg kg^− 1^, the likelihood of excellent intubating conditions was significantly decreased (ARD − 22% to − 67%). With 0.3 and 0.4 mg kg^− 1^, but not with 0.5, 0.6, 0.8, 1.5 and 2.0 mg kg^− 1^, the likelihood of unacceptable intubating conditions was significantly increased (ARD + 22% and + 32%, respectively). With 2.0 mg kg^− 1^, but not with 0.8 or 1.5 mg kg^− 1^, the likelihood of excellent intubating conditions was significantly increased (ARD + 23%). Apnoea times were significantly shorter with regimens ≤0.8 mg kg^− 1^ (MD − 1.0 to − 3.4 min) but were not reported with 1.5 or 2.0 mg kg^− 1^.

**Conclusions:**

With succinylcholine regimens ≤0.5 mg kg^− 1^, excellent intubating conditions are less likely and apnoea times are shorter, compared with 1 mg kg^− 1^. With 0.3 and 0.4 mg kg^− 1^, unacceptable intubating conditions are more common. Succinylcholine 1.5 mg kg^− 1^ does not produce more often excellent conditions compared with 1 mg kg^− 1^, while 2.0 mg kg^− 1^ does, but the database with these regimens is weak and apnoea times remain unknown. Limited information size and strong statistical heterogeneity decrease the certainty of the evidence.

## Background

Succinylcholine, also known as suxamethonium, has been introduced into anaesthesia practice in the early 1950s [1]. Still today, it remains one of the most commonly used neuromuscular blocking agents for rapid sequence induction (RSI) because of its fast onset and short duration of action [2]. The “cannot intubate, cannot ventilate” scenario is a threat of airway management. Therefore, clinicians are inclined to administer the minimally effective dose of succinylcholine that is meant to provide excellent intubating conditions but that provokes only a short apnoea time. The widely recommended standard intubating regimen of succinylcholine has been 1.0 mg kg^− 1^, although the scientific basis of that specific regimen remains unclear [3]. Indeed, a dose of 1.0 mg kg^− 1^ corresponds to almost four times the ED95, which is unusual for a neuromuscular blocking agent [4, 5].

We performed a systematic review and meta-analysis of randomized controlled trials (RCT) to determine the optimal regimen of succinylcholine for RSI. In this context, the optimal regimen was defined as the mg per kg bodyweight regimen that provided the highest likelihood of excellent intubating conditions, the lowest risk of unacceptable intubating conditions, and the shortest apnoea time compared with the gold standard regimen. As 1.0 mg kg^− 1^ has been reported to be the gold standard in this context [3], we compared all alternative, experimental regimens with that gold standard regimen.

## Methods

We performed a quantitative systematic review following the guidelines of the Preferred Reporting Items for Systematic Reviews and Meta-Analyses (PRISMA) statement and according to Cochrane methodology [6, 7]. A PRISMA checklist is available as a supplement (Table S1).

### Systematic search and study selection

Two authors (MG, AP) searched three electronic databases (PubMed, CENTRAL, EMBASE) from inception to 15 February 2019 using a variety of high-sensitivity and low-specificity search strategies. Details of the search strategy for PubMed are available as a supplement (Methods S1). There was no language restriction. Reference lists of retrieved articles were checked for further potentially relevant publications (backward snowballing). Retrieved articles were screened by three authors (CC, AP, MG). Discrepancies and queries for inclusion were resolved through consensus. If agreement could not be reached, discrepancies were discussed with the fourth author (MRT).

### Inclusion and non-inclusion criteria

We included full reports of RCTs performed in adults (≥18 years) undergoing surgery that compared any experimental regimen of succinylcholine with the standard regimen, 1.0 mg kg^− 1^, for RSI. We included studies that tested a ‘true’ or a ‘modified’ RSI procedure [8]. A true procedure involves intravenous induction with a hypnotic, intravenous administration of succinylcholine immediately (i.e. without any delay) after loss of consciousness, an apnoea period of no more than 60 s followed by orotracheal intubation. A modified RSI procedure is different in that the delay between loss of consciousness and the administration of the neuromuscular blocking agent is longer and allows, for instance, the additional recording of electromyographic baseline measures. During this time period, the patient is usually ventilated and oxygenated through a facemask. However, as with true RSI, after administration of the neuromuscular blocking agent, the apnoea period before orotracheal intubation is lasting no longer than 60 s.

Eligible trials had to report on intubating conditions using a validated score that evaluated ease of laryngoscopy, vocal cord position and movement, airway reaction, and movement of limbs [9]. We did not consider studies including obese patients for two reasons. Firstly, obesity is an independent risk factor of difficult laryngoscopy and tracheal intubation [10–12]. Secondly, the best succinylcholine regimen in obese patients remains controversial [13, 14]. Data from non-randomized trials, paediatric studies, abstracts, and trials that lacked a succinylcholine 1.0 mg kg^− 1^ group were also not taken into account.

### Data extraction

Two authors (MG, CC) read the full-text articles, extracted independently all relevant information and entered the data into a predefined electronic form. Discrepancies were resolved by discussion with a third author (MRT).

### Outcomes

According to good clinical research practice in pharmacodynamic studies of neuromuscular blocking agents [9], excellent, good or unacceptable intubating conditions may be distinguished. Excellent intubating conditions are present when all variables of the intubating score (ease of laryngoscopy, vocal cord position and movement, airway reaction, movement of limbs) are rated as excellent. Unacceptable intubating conditions are present when at least one variable of the intubating score is rated as poor. We chose the incidence of excellent intubating conditions (evaluated 50 to 60 s after the administration of succinylcholine) as the primary outcome as we regarded this outcome as the clinically most relevant in the context of RSI. The incidence of unacceptable intubating conditions was regarded as a secondary outcome. Good intubating conditions were not further analysed as we did not expect these data to inform decision-making. A further secondary outcome was apnoea time. Two definitions of apnoea time were used in the retrieved trials. First, apnoea time was defined as the time in minutes from succinylcholine administration to the occurrence of the first visible diaphragmatic contractions that coincided with movements of the reservoir bag. It was shown that this definition correlated with the incidence of haemoglobin desaturation defined by an oxygen saturation less than 80% [15]. Second, in some trials, apnoea time was defined as obvious recognizable end-tidal CO_2_ waveforms appearing on the monitor.

### Risk of Bias in individual studies

Quality of data reporting was assessed by two authors (MG, CC) and independently checked by another author (AP) using the Cochrane Collaboration method [7] and a modified 4-item, 7-point Oxford scale taking into account the method of randomization, concealment of treatment allocation, degree of blinding, and reporting of drop-outs, as previously described [16]. In the case of divergence of opinion, consensus was reached by discussion with the fourth author (MRT).

### Statistical analyses

Many comparisons contained zero cells, which made the calculation of risk ratios impossible. In order not to lose potentially relevant information, we decided to calculate absolute risk differences (ARD) with 95% confidence intervals (CI) for dichotomous data. When the 95% CI around the ARD did not cross 0, the result was considered statistically significant (*p* value equal or inferior to 0.05). We also computed numbers needed to treat (NNT) with 95% CI as the inverse of the ARD point estimates and the lower and upper limits of their 95% CI. The NNT was the estimated number of patients who needed to be treated with the experimental regimen for one additional patient to have one more outcome compared with the comparator. A positive ARD suggested that an outcome was improved with an experimental regimen compared with the standard regimen and was consequently translated into a positive NNT. A negative ARD suggested that an outcome was worsened with an experimental regimen compared with the standard regimen and was consequently translated into a negative NNT (which may then be interpreted as a “number needed to harm”). An ARD of 0, indicating no difference between the experimental and the standard regimen, was translated into an NNT of infinity (∞). For continuous outcomes, we computed mean differences (MD) with 95% CI. We used a random-effects model throughout (Mantel-Haenszel method). Between studies heterogeneity was quantified using the I^2^ statistics. We performed sensitivity analyses computing relative instead of absolute risk differences and using a fixed-effect instead of a random effects model. Statistical analyses were performed with Review Manager (RevMan [Computer program], Version 5.3; The Nordic Cochrane Centre, The Cochrane Collaboration, Copenhagen, Denmark, 2014) and Microsoft Excel 2010 (for Mac).

## Results

### Study selection

**Fig. 1:**
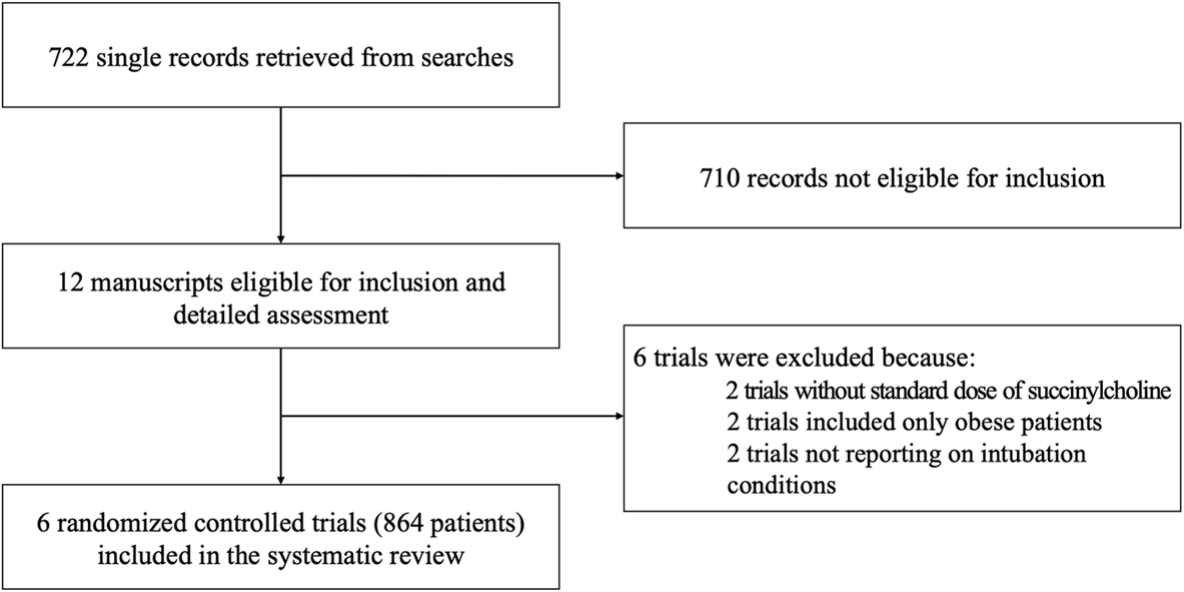
Flow diagram of the study selection process

Our searches yielded 722 potentially relevant reports (Fig. 1). After exclusion of 690 inappropriate studies, 12 studies were retrieved as complete articles. Of these, six were excluded as they did not meet the inclusion criteria. Two of those tested different doses of succinylcholine without comparison with the gold standard regimen (1 mg kg^− 1^) [17, 18], two included obese patients only (body mass index ≥40 kg m^− 2^) [13, 14], and two did not report on intubating conditions [19, 20]. We finally included six RCTs with relevant data on 864 patients (Fig. 1) [21–26].

### Study characteristics

Eligible studies were published between 2003 and 2014 (Table 1). They were performed in four countries (China, India, Saudi Arabia, USA) and included between 69 and 200 patients. Patients were ASA 1 or 2, 18 to 65 years old, with a body mass index < 30 kg m^− 2^. All patients were eligible for elective surgery under general anaesthesia with tracheal intubation, were fasting preoperatively, had no criteria of difficult airway, no contraindication for succinylcholine, and no family history of an abnormal response to succinylcholine.

**Table 1:** Characteristics of included trials

Trial	Country	Experimental regimens [mg kg^− 1^] (number of patients)	PM	Medications for induction	Rapid Sequence Induction	Cricoid pressure	Adverse effects	Intubation failures
El Orbany 2004	USA	0.3 (n = 23) 0.4 (*n* = 23) 0.5 (*n* = 23) 0.6 (*n* = 23) 1.0 (*n* = 23)	Yes	Fentanyl 1.5 μg kg^− 1^ Propofol 2 mg kg^− 1^	Modified	nr	nr	7 with succinylcholine 0.3 mg kg^− 1^; 2 with succinylcholine 0.4 mg kg^− 1^
Luo 2014	China	0.3 (*n* = 60) 0.5 (*n* = 60) 1.0 (*n* = 60)	No	Sevoflurane	True*	nr	“No complications such as cough, laryngospasm, or bronchial spasm attributable to the study”	None
Naguib 2003	Saudi Arabia	0.3 (*n* = 50) 0.5 (*n* = 50) 1.0 (*n* = 50)	Yes	Fentanyl 2 μg kg^− 1^ Propofol 2 mg kg^− 1^	True	nr	“Each patient was followed up for any adverse affects”. Results nr.	None
Naguib 2006	USA	0.3 (*n* = 30) 0.5 (*n* = 30) 1.0 (*n* = 30) 1.5 (*n* = 30) 2.0 (*n* = 30)	No	Fentanyl 2 μg kg^− 1^ Propofol 2 mg kg^− 1^	True	nr	“Each patient was monitored for any adverse event”. Results nr.	None
Prakash 2012	India	0.4 (*n* = 23) 0.6 (*n* = 23) 1.0 (*n* = 23)	No	Fentanyl 2 μg kg^− 1^ Propofol 2 mg kg^− 1^	True	yes	Adverse events were recorded and no episodes of laryngospasm, bronchospasm, masseter spasm, generalized rigidity were observed	None
Taxak 2013	India	0.4 (*n* = 50) 0.6 (*n* = 50) 0.8 (*n* = 50) 1.0 (*n* = 50)	Yes	Meperidine 1 mg kg^− 1^ Propofol 2 mg kg^− 1^	Modified	nr	nr	nr

*PM* premedication, *nr* not reported, ^a^true RSI with sevoflurane

All trials included a group with a standard succinylcholine regimen (1.0 mg kg^− 1^). Experimental regimens were 0.3 mg kg^− 1^ [21–23, 26], 0.4 mg kg^− 1^ [22, 24, 25], 0.5 mg kg^− 1^ [21–23], 0.6 mg kg^− 1^ [22, 24–26], 0.8 mg kg^− 1^ [25], 1.5 mg kg^− 1^ [23], and 2.0 mg kg^− 1^ [23]. Five trials were double blinded. The modified Oxford quality score ranged from 2 to 6. One trial was not blinded and therefore judged to be at high risk of bias (Figure S1 and S2) [23]. Two trials reported sources of authors’ funding and conflicts of interest [24, 26].

### Induction techniques

In three studies, patients were premedicated (Table 1) [21, 23, 25]. Three studies used a true RSI [21, 23, 24], and two a modified RSI [22, 25]. All five used intravenous propofol 2 mg kg^− 1^ with a concomitant intravenous opioid for induction. The sixth study used an induction technique with sevoflurane without intravenous opioids; there was no delay between loss of consciousness and the administration of succinylcholine [26]. One study specified the use of a cricoid pressure [24]. In all studies, intubating conditions were evaluated 50 to 60 s after the administration of succinylcholine. Four studies specified that intubations were performed by an experienced anaesthetist [21–24].

### Quality of intubating conditions

#### Excellent intubating conditions

**Fig. 2:**
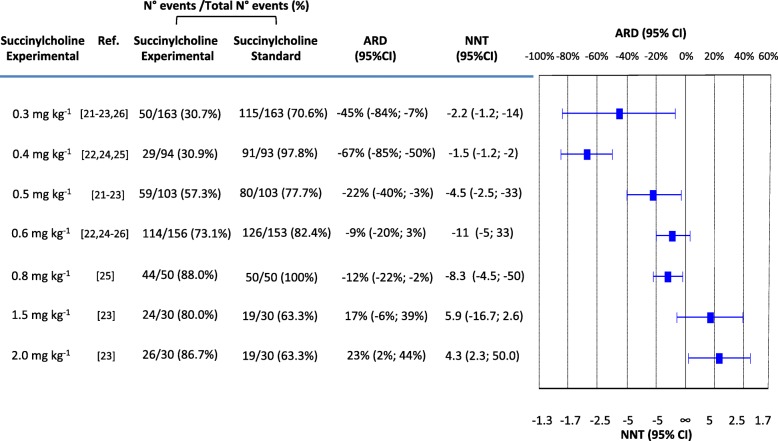
Excellent intubating conditions with the standard regimen of succinylcholine (1.0 mg kg^− 1^) compared with different experimental regimens. Comparisons are listed according to increasing experimental doses. ARD = absolute risk difference; NNT = number needed to treat; CI = confidence interval; ∞ = infinity (i.e. ARD = 0). A positive ARD suggested that an outcome was improved with an experimental regimen compared with the standard regimen and was consequently translated into a positive NNT. A negative ARD suggested that an outcome was worsened with an experimental regimen compared with the standard regimen and was consequently translated into a negative NNT (which may be interpreted as a “number needed to harm”). An ARD of 0, indicating no difference between the experimental and the standard regimen, was translated into an NNT of infinity (∞)

With the standard succinylcholine regimen (1.0 mg kg^− 1^), the incidence of excellent intubating conditions ranged from 58% [26] to 100% [24, 25] (cumulative number of patients, 185 of 233 [79%]) (Fig. 2).

With four experimental regimens that were tested in at least three trials each (0.3, 0.4, and 0.5 mg kg^− 1^) [21–26], excellent intubating conditions were significantly less frequent compared with 1.0 mg kg^− 1^ (ARD ranging from − 9% to − 67%) (Fig. 2). With 0.8 mg kg^− 1^, tested in one trial only [25], excellent intubating conditions were also significantly less frequent compared with 1.0 mg kg^− 1^ (ARD − 12%) (Fig. 2). With 0.6 and 1.5 mg kg^− 1^, no difference was found. With 2.0 mg kg^− 1^, excellent intubating conditions were significantly more frequent compared with 1.0 mg kg^− 1^ (ARD + 23%) (Fig. 2). Both 1.5 and 2 mg kg^− 1^ were tested in one trial only [23]. The I^2^ ranged from 54% to 95% (Figure S3).

#### Unacceptable intubating conditions

**Fig. 3:**
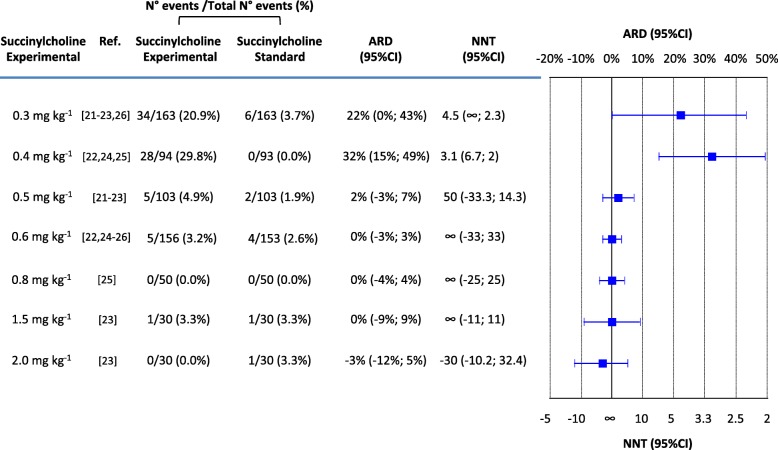
Unacceptable intubating conditions with the standard regimen of succinylcholine (1.0 mg kg^− 1^) compared with different experimental regimens. Comparisons are listed according to increasing experimental doses. ARD = absolute risk difference; NNT = number needed to treat; CI = confidence interval; ∞ = infinity (i.e. ARD = 0). A positive ARD suggested that an outcome was improved with an experimental regimen compared with the standard regimen and was consequently translated into a positive NNT. A negative ARD suggested that an outcome was worsened with an experimental regimen compared with the standard regimen and was consequently translated into a negative NNT (which may be interpreted as a “number needed to harm”). An ARD of 0, indicating no difference between the experimental and the standard regimen, was translated into an NNT of infinity (∞)

With the standard succinylcholine regimen (1.0 mg kg^− 1^), the incidence of unacceptable intubating conditions ranged from 0% [22, 24, 25] to 6.7% [26] (cumulative number of patients, 6 of 233 [2.6%]) (Fig. 3). With 0.3 and 0.4 mg kg^− 1^, tested in at least three trials each [21–25], unacceptable intubating conditions were significantly more frequent compared with 1.0 mg kg^− 1^ (ARD + 22% and + 32%) (Fig. 3). With 0.5 and 0.6 mg kg^− 1^, also tested in at least three trials each [21–25], and with 0.8, 1.5 and 2.0 mg kg^− 1^, tested in one trial each [23, 25], the likelihood of unacceptable intubating conditions was no different from 1.0 mg kg^− 1^ (Fig. 3). The I^2^ ranged from 0% to 93% (Figure S4).

#### Apnoea times

**Fig. 4:**
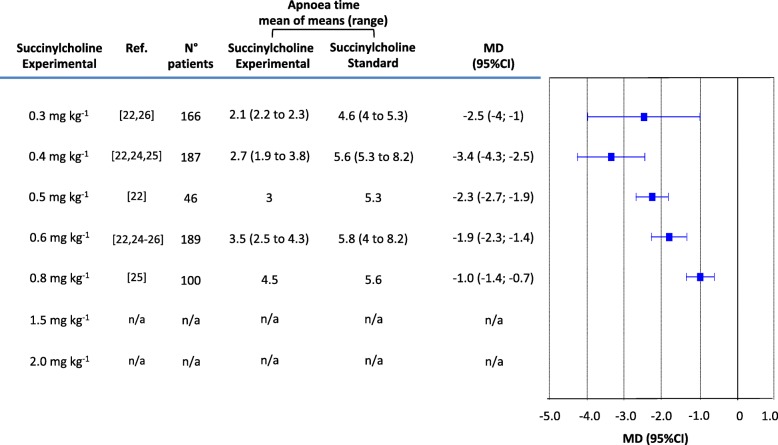
Apnoea times (in min) with the standard regimen of succinylcholine (1.0 mg kg^− 1^) compared with different experimental regimens. The time from injection of succinylcholine until first diaphragmatic movement or until obvious recognizable end-tidal CO2 waveforms appearing on the monitor was used as a surrogate of apnoea time. MD = mean difference; CI = confidence interval; n/a = not applicable (no data reported)

With the standard succinylcholine regimen (1.0 mg kg^− 1^), average apnoea times, reported in four trials [22, 24–26], ranged from 4.0 min [26] to 8.2 min [24] (Fig. 4). Two experimental regimens, 0.4 and 0.6 mg kg^− 1^, were tested in at least three trials [22, 24–26], and in both, apnoea times were significantly shortened compared with the gold standard regimen (MD, − 3.4 and − 1.9 min, respectively). Three regimens (0.3, 0.5, 0.8 mg kg^− 1^) were tested in one or two trials each [22, 25, 26] and were associated with shorter apnoea times compared with 1 mg kg^− 1^ (Fig. 4). With 1.5 and 2.0 mg kg^− 1^, no apnoea times were reported. The I^2^ ranged from 74% to 97% (Figure S5).

#### Sensitivity analyses

Computing risk ratios did not change the magnitude of the results (Figure S6), as did the use of a fixed effect model (Figure S7). The exclusion of one trial [22] decreased the degree of heterogeneity in some analyses but aggregated results remained similar (Figure S8).

## Discussion

### Main findings

We performed a systematic review and meta-analysis of RCTs to evaluate intubating conditions and apnoea times with different succinylcholine regimens. As a gold standard, we have chosen succinylcholine 1.0 mg kg^− 1^, which is probably the most widely used regimen for RSI. We analysed data from six trials including relevant data of 864 patients.

If we consider the outcome “excellent intubating conditions” as the most relevant in the context of RSI (Fig. 2), we may conclude that regimens equal or lower than 0.5 mg kg^− 1^ produced less often excellent conditions, whereas 2.0 mg kg^− 1^ performed significantly better that 1 mg kg^− 1^. With succinylcholine 2.0 mg kg^− 1^, the number needed to treat suggested that four to five patients needed to be intubated for one to have excellent intubating conditions during RSI who would not have had such excellent conditions had they all received 1 mg kg^− 1^ only. This result must be interpreted cautiously mainly for two reasons. Firstly, 2.0 mg kg^− 1^ was tested in one single trial only with a limited number of patients [23]. Secondly, in that single trial, only 63% of patients had excellent intubating conditions with the standard regimen, 1.0 mg kg^− 1^ [23]. This “baseline” incidence was lower compared with the other trials. It can, therefore, not be excluded that 2.0 mg kg^− 1^ performed well since in the only trial that tested this regimen [23], the gold standard regimen performed relatively badly. Thus, it remains unknown whether 2.0 mg kg^− 1^ further improves “excellent intubating conditions” in a patient population with a high baseline incidence of “excellent intubating conditions”.

Alternatively, the outcome “unacceptable intubating conditions” may be regarded as the most relevant in the context of RSI (Fig. 3). Contrary to the outcome “excellent intubating conditions”, the result was much more dichotomous; regimens below 0.5 mg kg^− 1^ were clearly less efficacious compared with the gold standard, 1 mg kg^− 1^, whereas regimens above 0.5 mg kg^− 1^ were no different from 1 mg kg^− 1^. Based on this outcome, it may be deduced that regimens below 0.5 mg kg^− 1^ should be avoided for RSI. Interestingly, with the gold standard regimen 1 mg kg^− 1^, the incidence of “unacceptable intubating conditions” showed much less variability between trials (0% to 6.7%) compared with the outcome “excellent intubating conditions”. This suggests that indirect comparisons between different succinylcholine regimens are more reliable when the outcome “unacceptable intubating conditions” is chosen. It also suggests that the outcome “unacceptable intubating conditions” is not ideal to test various degrees of efficacy of experimental regimens compared with the gold standard regimen; with regimens above 0.6 mg kg^− 1^, unacceptable intubating conditions were virtually absent.

Additionally to the outcomes “excellent” and “unacceptable” intubating conditions, apnoea time may be used for rational decision-making (Fig. 4). With regimens below 0.8 mg kg^− 1^, median apnoea times became constantly shorter, and consequently, mean differences compared with the gold standard regimen increased. There is thus an argument in favour of using lower than standard succinylcholine regimens for RSI. This must however be weighted against the increased risk of having less often excellent intubating conditions and more frequently unacceptable intubating conditions. Data on apnoea times with regimens above 1 mg kg^− 1^ were lacking. Thus, clinically relevant prolongation of apnoea times with 1.5 or 2.0 mg kg^− 1^ needs to be formally shown. Also, the primary factor determining return of spontaneous respiration may not be the depth of neuromuscular block, but the degree of centrally mediated respiratory depression induced by the opioids and hypnotics used for induction of anaesthesia.

### Strengths and limitations

The strength of this meta-analysis is the rigorous systematic review of the literature. Also, we included exclusively RCTs comparing different experimental regimens of succinylcholine with the gold standard regimen, 1 mg kg^− 1^, for RSI and using the same validated intubation score. However, the number of retrieved valid studies was small, the number of included patients limited, and statistical heterogeneity was relatively high. Thus, the evidence base remains weak and the interpretation of the data is not straightforward. Additionally, all trials were performed in low risk patients undergoing elective surgery, although succinylcholine is mainly used for RSI in patients undergoing emergency surgery. Some studies were using a modified RSI technique and in one [26], sevoflurane was used for induction. This may have introduced clinical heterogeneity. The observed variability of the baseline incidences of excellent intubating conditions suggests that the study populations were not similar or that intubation scores were interpreted differently. Finally, succinylcholine-related adverse effects were not systematically reported and for regimens above 1.0 mg kg^− 1^, data on apnoea times were lacking. For rational decision-making, adverse effects and apnoea times with all tested regimens should be known.

### Research agenda

It is surprising that efficacy and potential of harm of a drug that has been widely used for almost 60 years in the perioperative setting including anaesthesia, intensive care and emergency medicine, is so poorly documented. This begs the question as to the need for further high quality trials to better understand the role of succinylcholine in patients needing RSI. Specific high-risk patient populations have not be studied, including pregnant women, patients undergoing emergency surgery, and children. Also, the optimal succinylcholine regimen in obese patients remains controversial [13, 14], and needs further investigation. Trials should report on drug-related adverse effects and apnoea times.

## Conclusions

With succinylcholine regimens ≤0.5 mg kg^− 1^, excellent intubating conditions are less likely compared with the gold standard regimen, 1 mg kg^− 1^. With 0.3 and 0.4 mg kg^− 1^, unacceptable intubating conditions are more common. With regimens below 1 mg kg^− 1^, apnoea times are shorter. With 2 mg kg^− 1^, excellent intubating conditions seem to be more likely but the database is weak, and apnoea times remain unknown. Small information size and strong statistical heterogeneity limit the certainty of the evidence.

## Supplementary information

**Additional file 1: Table S1.** PRISMA 2009 Checklist. **Methods S1**. Search strategy for PubMed. **Figure S1.** Risk of bias graph: review authors’ judgments about each risk of bias item presented as percentages across all included studies. **Figure S2.** Risk of bias summary: review authors’ judgments about each risk of bias item for each included study. **Figure S3.** Excellent intubating conditions. **Figure S4.** Unacceptable intubating conditions. **Figure S5.** Apnea times. **Figure S6.** Sensitivity analyses - Fixed effects model meta-analysis. **Figure S7.** Sensitivity analyses - Random effects model meta-analysis with relative risk. **Figure S8.** Sensitivity analyses - Meta-analysis excluding El-Orbany 2004.

## Data Availability

the datasets used and/or analysed during the current study are available from the corresponding author on reasonable request.

## Abbreviations

ARD: Absolute risk differences
CI: Confidence interval
MD: Mean difference
NNT: Number needed to treat
PRISMA: Preferred Reporting Items for Systematic Reviews and Meta-Analyses
RCT: Randomized controlled trial
RSI: Rapid sequence induction
